# Managing errors in the eye unit

**Published:** 2019-09-10

**Authors:** Larry Benjamin, David Yorston, John Buchan

**Affiliations:** 1 Consultant Ophthalmologist: Stoke Mandeville Hospital, Aylesbury, UK.; 2 Consultant Ophthalmologist: Tennent Institute of Ophthalmology, Gartnavel Hospital, Glasgow, Scotland, UK.; 3 Consultant Ophthalmologist: International Centre for Eye Health, London School of Hygiene and Tropical Medicine, UK.

**It takes a team to deliver eye care. When things go wrong, it is important to focus on the patient and to learn from the error while working supportively with the health professionals involved.**

**Figure:**
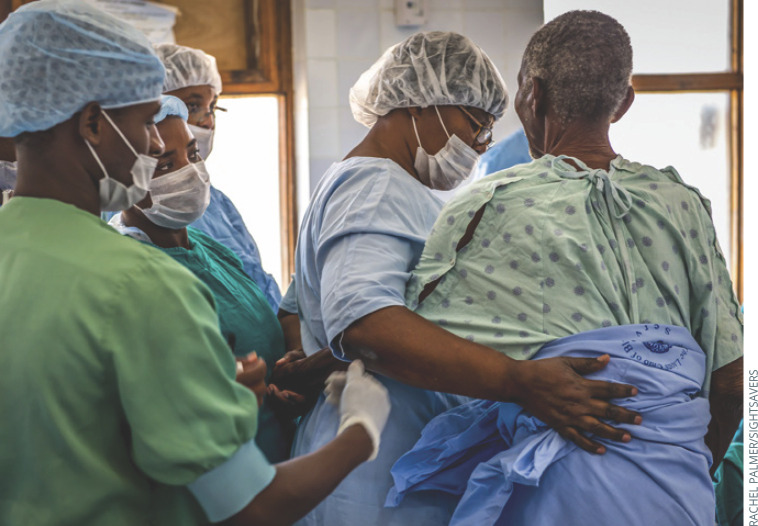
Caring for the patient is the main responsibility of an eye health worker, especially if something has gone wrong. MALAWI

No-one working in eye care wants to make a mistake. To protect our patients, it is vital that we anticipate problems and develop systems that minimise risk, such as the WHO Guidelines on Safe Surgery and the WHO Surgical Safety Checklist.^1^

If something goes wrong, however, we have three main responsibilities:

1. **Care for the patient.** Be honest, tell them that something has gone wrong (p. 21) and provide appropriate care.
2. **Support the health worker involved** (including training).
3. **Learn from the error**, so it does not happen again.

## 1. Care for the patient

### Ensure that harm to the patient is minimised

For instance, contact the patient immediately if you find out that there has been a drug error. Find out whether the patient has been harmed, and deal with any consequences of the error.

As an example, if you discover that a dilution error has occurred in the preparation of antibiotics for an intra-ocular injection, immediately contact all the patients involved to see if they are symptomatic. Offer to examine them and treat any adverse reactions.

### Apologise to the patient(s)

Never be afraid to apologise to the patient(s) concerned. This is not an admission of liability, but an expression of sympathy, and most people would expect it (p. 21).

### Avoid cover-ups and promote a ‘no blame’ culture

It is very important for managers and senior colleagues to promote a culture of openness and transparency, free from fear of blame or retribution, so that issues can be openly discussed and a way forward found.

A ‘no blame’ culture (see panel on p. 27) means that staff members feel free to report incidents immediately, rather than covering them up until more harm is caused.

## 2. Support the health workers involved

Most health workers will be deeply affected by their involvement in an event that harms a patient. They are sometimes referred to as the ‘second victims’ of medical errors, as involvement in a medical error can lead to a loss of confidence, and they may even leave the profession. We cannot afford to lose skilled health workers, so it is important that they are rehabilitated as well.

### Include health workers in the process of patient recall, assessment and treatment

Health workers benefit when their involvement with a patient continues after an error is discovered, e.g., by arranging an appointment and examining them, as it gives them an opportunity to do something positive for the patient. By asking the health worker to remain involved, you are showing that you still have confidence in them, which will help to rebuild their confidence in themselves. Finally, being involved in an error provides a valuable opportunity for them to learn, with the support of their colleagues, how to handle the situation in future.

### Encourage (healthy) reflection

Invite health workers to think about what happened, and why. Did they follow the correct procedure? Were they tired? Was there a communication error, and why? Are there adequate systems in place to minimise the risk of error?

Health workers may blame themselves, even when it is not their fault. If they think the error is their fault, ask whether this is a realistic assessment. Would another reasonable, trained person have acted differently if they had access to the same information at the time?

The converse may also be true. Some health workers may be unwilling to accept any responsibility, instead blaming everyone else without considering their own role in the event. These health workers may well repeat their error, which makes them dangerous to patients. Health workers who engage constructively and accept that they may have played a part in an error are less likely to repeat the same mistakes.

### Involve health workers in prevention

Ask questions such as: “What suggestions do you have to help us avoid this sort of mistake in the future?” or, “Can you see any steps in the handling of medicines that might need to be changed to prevent this error occurring again?”

This is vital, as the health worker may have insights that more senior members of the team cannot understand. For example, senior doctors and managers rarely dispense eye drops. If there is a problem with the dispensing system, the more junior members of the team are far more likely to know about it.

## 3. Learn from incidents

It is also good practice to make reporting all such incidents routine, for instance with a departmental “incident book”. Incidents can then be regularly discussed at staff meetings, and lessons learned to improve standards and outcomes.

Investigate how the error was made, and make sure that accurate information is obtained (e.g., patient notes, the prescription and dispensing instructions) so that the issue can be discussed, in a constructive way, with all those concerned.

Identify whether there are any gaps in staff members' training, and address these by offering relevant training, supervision, or teaching so that a similar error is not repeated in the future.

Sometimes, an error is brought to light as a result of a complaint being made by a patient or, possibly, another staff member. Unless it is felt to be a matter that may immediately affect patient safety, it is important to hear and investigate both sides of the story before taking any action. Organise a meeting with the health worker(s) concerned as soon as possible.

**How to break bad news**

It is essential to provide formal teaching and/or guidelines on breaking bad news; this is invaluable as it gives everyone a starting place when trying to address these situations. This can be done by role play, but it is also vital that health workers in training can observe senior staff giving bad news in real life.

In our hospital, the health worker(s) involved will tell the patient that a mistake has been made, and that:

- They are deeply sorry that this has happened
- Everything possible will be done to help sort out the problem and manage any complications
- Senior members of the team will be involved to ensure that the best possible outcome is reached.

This reassures the patient that everything possible is being done to resolve the problem and that they will get the support and treatment they need. This can also help other health care workers feel reassured that they will get the support of their team and management if, or when, they are involved in an error.

## Leadership

It is important that leaders work with eye teams to:

- Make patient safety a priority
- Audit errors regularly, and make someone responsible for this
- Put a clear process in place to address medical errors
- Create a no-blame culture and a supportive working environment – this should help to prevent health workers feeling as if they need to cover up their mistakes (p. 36)
- Ensure that everyone can learn from the incident, so that it is not repeated.
